# Comparison of Support Vector Machine, Naïve Bayes and Logistic Regression for Assessing the Necessity for Coronary Angiography

**DOI:** 10.3390/ijerph17186449

**Published:** 2020-09-04

**Authors:** Parastoo Golpour, Majid Ghayour-Mobarhan, Azadeh Saki, Habibollah Esmaily, Ali Taghipour, Mohammad Tajfard, Hamideh Ghazizadeh, Mohsen Moohebati, Gordon A. Ferns

**Affiliations:** 1 Department of Epidemiology and Biostatistics, School of Health, Mashhad University of Medical Sciences, Mashhad 917791-8564, Iran; parastoogolpour@gmail.com; 2 International UNESCO Center for Health-Related Basic Sciences and Human Nutrition, Mashhad University of Medical Sciences, Mashhad 917791-8564, Iran; ghayourm@mums.ac.ir (M.G.-M.); ghazizadehh2@gmail.com (H.G.); 3 Cardiovascular Research Center, Faculty of Medicine, Mashhad University of Medical Sciences, Mashhad 917791-8564, Iran; mouhebatim@mums.ac.ir; 4 Social Determinants of Health Research Center, Mashhad University of Medical Sciences, Mashhad 917791-8564, Iran; esmailyh@mums.ac.ir (H.E.); TaghipourA@mums.ac.ir (A.T.); TajfardM@mums.ac.ir (M.T.); 5 Department of Epidemiology, School of Health, Mashhad University of Medical Sciences, Mashhad 917791-8564, Iran; 6 Department of Health Education and Health Promotion, Faculty of Health, Mashhad University of Medical Sciences, Mashhad 917791-8564, Iran; 7 Student Research Committee, Mashhad University of Medical Sciences, Mashhad 917791-8564, Iran; 8 Brighton & Sussex Medical School, Division of Medical Education, Falmer, Brighton, Sussex BN1 9PH, UK; g.ferns@bsms.ac.uk

**Keywords:** logistic regression, support vector machine, naïve Bayes, angiography

## Abstract

(1) Background: Coronary angiography is considered to be the most reliable method for the diagnosis of cardiovascular disease. However, angiography is an invasive procedure that carries a risk of complications; hence, it would be preferable for an appropriate method to be applied to determine the necessity for angiography. The objective of this study was to compare support vector machine, naïve Bayes and logistic regressions to determine the diagnostic factors that can predict the need for coronary angiography. These models are machine learning algorithms. Machine learning is considered to be a branch of artificial intelligence. Its aims are to design and develop algorithms that allow computers to improve their performance on data analysis and decision making. The process involves the analysis of past experiences to find practical and helpful regularities and patterns, which may also be overlooked by a human. (2) Materials and Methods: This cross-sectional study was performed on 1187 candidates for angiography referred to Ghaem Hospital, Mashhad, Iran from 2011 to 2012. A logistic regression, naive Bayes and support vector machine were applied to determine whether they could predict the results of angiography. Afterwards, the sensitivity, specificity, positive and negative predictive values, AUC (area under the curve) and accuracy of all three models were computed in order to compare them. All analyses were performed using R 3.4.3 software (R Core Team; Auckland, New Zealand) with the help of other software packages including receiver operating characteristic (ROC), caret, e1071 and rminer. (3) Results: The area under the curve for logistic regression, naïve Bayes and support vector machine were similar—0.76, 0.74 and 0.75, respectively. Thus, in terms of the model parsimony and simplicity of application, the naïve Bayes model with three variables had the best performance in comparison with the logistic regression model with seven variables and support vector machine with six variables. (4) Conclusions: Gender, age and fasting blood glucose (FBG) were found to be the most important factors to predict the result of coronary angiography. The naïve Bayes model performed well using these three variables alone, and they are considered important variables for the other two models as well. According to an acceptable prediction of the models, they can be used as pragmatic, cost-effective and valuable methods that support physicians in decision making.

## 1. Introduction

Cardiovascular disease (CVD) is the most common cause of death in most countries, including Iran, and the most important cause of disability. Approximately 17.5 million people die of CVD each year [1]. In spite of the improvement in diagnosis and treatment, one-third of patients suffering from myocardial infarction die, and two-thirds of those who survive will never recover completely. This imposes a significant cost on health systems [2].

CVD is a disease that affects the heart or blood vessels. Coronary artery disease (CAD) is the most common type of this class. CAD results when the arteries that supply blood to heart muscle become hardened and narrowed. As a result, the heart muscle cannot receive the blood or oxygen it needs. This can lead to chest pain (angina), or a heart attack (myocardial infarct). A heart attack occurs when at least one coronary artery is completely blocked [3].

Angiography is the most reliable method among the various methods of diagnosis for CAD. However, angiography is a costly and invasive procedure [3]. Moreover, various studies have shown that angiography may result in complications [4]. Thus, it is important to use methods that may increase the accuracy of diagnostic and therapeutic tests such as angiography.

Machine learning is considered to be a branch of artificial intelligence. Its aims are the design and development of algorithms that give permission to computers for improving their performances with data. The process involves the analysis of past experiences to find practical and helpful regularities and patterns that a human might overlook. The development of automatic models is a central focus of machine learning research, for instance, rules and patterns extracted from data. Learning patterns contribute to machine learning and are derived from animal and human learning patterns, but can now be used to teach us how animals and humans learn [5].

Medical and computer scientists have worked together for several years, and data mining has allowed the extraction of worthwhile information from large data sets. It is possible that data mining tools could be applied as an alternative to using expensive and invasive medical procedures [5].

In clinical management, tests are often applied to establish the presence or absence of disease. Therefore, for confirming the presence of cardiovascular disease such as coronary heart disease and stroke, the patients may undergo several tests (biochemical tests, rest electrocardiogram (ECG), echocardiography, stress tests or angiography). Some are invasive, uncomfortable for patients, expensive and time consuming. Data mining techniques are capable of identifying high-risk patients to define the most important variables in cardiovascular patients. They also have the ability to allow the building of a risk model [5].

Presently, data mining and machine learning have attracted attention in the prediction and diagnosis of disease, however, these methods are very complex, and require a high level of experience and knowledge. However, their application may reduce medical errors and improve the quality of diagnosis. Logistic regression, support vector machine and naïve Bayes are the most widely used models.

A logistic regression is a generalized linear model with canonical link functions. A logistic regression is used when the dependent variable is categorical, which is the most common model for binary response data. This model calculates the probability of events by using a linear function of the values of explanatory variables. Furthermore, having no limitation for these explanatory variables is considered a benefit of this model [6,7,8].

A support vector machine is considered to be an extension of the maximum margin classification, because it is able to classify nonlinear classifications by the selection of a line with maximum-margin with the help of some points called support vectors. The support vector machine model allows us to increase the feature space using the kernel functions so that the computations are efficient and manageable.

The naïve Bayes method is based on Bayes’ theorem. In this method, the probability that a multiplicity X (*x*_1_, *x*_2_, …, *x*_n_) belongs to a category is measured. Bayes’ theorem makes it possible to calculate the posterior probability based on the prior probabilities.

The computation of this model is simple since it is assumed that the value of a particular feature is independent of the value of any other feature [9].

Therefore, the aim of this study was to investigate and compare the support vector machine, naïve Bayes and logistic regression to determine the diagnostic factors for the necessity of angiography.

## 2. Materials and Methods

This cross-sectional study was performed on 1187 candidates referred for angiography to Ghaem Hospital in Mashhad, Iran from 2011 to 2012. The study was approved by the Mashhad University of Medical Sciences Ethics Committee (reference number 900671). A positive coronary angiogram is usually defined as having an obstruction greater than 50% of at least one coronary artery, and a negative angiogram indicates an obstruction of less than 50% of any coronary artery.

Patients over 18 years of age, who were candidates for the angiography according to a specialist cardiologist, and who agreed to participate in the study, were enrolled.

Patients with the following conditions were excluded from the study: illness such as kidney or chronic liver disease, rheumatism, immune deficiency disease, any type of cancer, inflammatory disease such as inflammatory bowel disease, infectious disease in the last three months, history of any type of surgery in the last three months, history of coronary angioplasty, taking drugs such as steroids, penicillin, oral contraceptives, hormone replacement therapy, pregnancy and lactation.

Two checklists were used to assess variables that included information on patients’ medical records and laboratory results. The variables included in this study were: age, gender, marital status, levels of education, smoking, high fat, history of cardiovascular disease, history of myocardial infarction, family history of cardiovascular disease, family history of kidney disease, family history of hypertension, fasting blood glucose (FBG), serum TGs (triglycerides), high blood pressure, serum HDL (high-density lipoprotein), and the results of angiography.

### Statistical Models

Logistic regression, support vector machine and naïve Bayes models were used in this research to predict the necessity of angiography. A logistic regression is a generalized linear model with a canonical link function. In terms of easy computation, a logistic regression is the best model among generalized linear models for binary response data. Interpreting the result of this model is very simple and logical in medical studies because of using odds instead of risk in the link function [6,10].

(1) $$logit\left(p\right)=\ln\left(\frac{p}{1-p}\right)=\propto +\beta x$$

P = The success probability at value *x*
b = Determines the rate of change; when b > 0, *p* increases as *x* increases and when b < 0, *p* decreases as *x* increases
a = Value of *p* when b = 0

A support vector machine is one of the supervised learning models used to classify data. The aim of an Support Vector Machine (SVM) is to perform a linear classification and also select a line with a maximum-margin.

The SVM attempts to select a decision boundary to maximize the minimum distance to each of the categories. SVM is robust in the presence of noise. The process of selecting the best decision boundary is based on some points called support vectors.

The aim of an SVM is to maximize the margin. The following formulae help us to reach this aim.

Margin = 2||w||^2

W: normal vector of the hyperplane; the normal vector is perpendicular to the both hyperplanes which are parallel to each other; therefore, with the help of a normal vector we can calculate the distance between the two hyperplanes. In SVMs, the aim is to choose w in a way that it can maximize the distance between hyperplanes.

Maximizing the margin is equivalent to minimizing the following formula:

L(W) = ||W||^22

Moreover, an SVM could efficiently perform a nonlinear classification by using the kernel functions that map data into high-dimensional feature spaces. The radial kernel function used in this paper takes the form

$$K\left(x_{i},x_{i^{\prime }}\right)=exp\left(-\gamma +\;\overset{p}{\underset{j=1}{\sum }}{\left(x_{ij}-x_{i\prime j}\right)}^{2}\right)$$

*γ* is a positive constant. If a given test observation x* = ($x_{1}^{*}$ … $x_{p}^{*}$), T is far removed from a training observation *x_i_* in terms of the Euclidean distance, then $\overset{p}{\underset{j=1}{\sum }}{\left(x_{j}^{*}-\;x_{ij}\right)}^{2}$ will be large, and so $k\left(x^{*},x_{i}\right)$=$\exp(-\overset{p}{\underset{j=1}{\sum }}{\left(x_{j}^{*}-x_{ij}\right)}^{2})$ will be very small. This means that in the nonlinear function

$$f\;(x)\;=\;x\beta_{0}+\sum^{\;}\propto_{i}k\;\left(x,x_{i}\right)$$

*x_i_* will have no effect on f(x*). The predicted class label for X* (test observation) is based on the sign of f(x*), so the training observations that are far from x* will not have any effect on predicting the class label for x*. In other words, the radial kernel exhibits local behavior because merely nearby training observations play a role in the predicted class label for a test observation [8].

The naïve Bayes method is based on Bayes’ theorem. This theorem makes it possible to calculate the posterior probability based on the prior probabilities.

P(c) and P(A) are the probabilities of observing A and C independently of each other; this is known as the marginal probability.

P (A|c) is a condition probability: the likelihood of event A occurring given that C is true

Bayes’ theorem: P (c|A) = $\frac{P\;(A|C)p\left(c\right)}{P\left(A\right)}$.

Firstly, p(C|A1,A2,A3, …, An) is calculated for all categories with the help of Bayes’ theorem.

P(C|A1,A2,A3, …, An) = $\;\frac{P\;\left(A1,A2,A3,\ldots ,\mathrm{An}|C\right)p\left(C\right)}{P\left(A1,A2,A3,\ldots ,\mathrm{An}\right)}$.

The category with the maximum value of p(C|A1,A2,A3, …, An) is selected. Since the denominator of the mentioned formula is the same for all categories, we aim to find the category with the maximum value of the numerator. On the other hand, the value that should be computed for each set of data is p(A1, A2, A3, …, An|C).

If the features are independent we have:

P (A1,A2,A3, …, An|Cj (=P (A1|Cj) P (A2|Cj) P (A3|Cj) …. P (An|Cj).

The probability of observing a category is calculated as follows:

P (C) = Nc/N;

N: total number of records;

Nc: number of records for category.

A sensitivity analysis was used to select the most important variables in naïve Bayes and SVM models. The variables that optimize the value of the maximum area under the curve were selected as final variables. In addition, since there was no significance difference between the SVM with polynomial and radial kernel functions, the latter that had fewer parameters was selected. Regarding the logistic regression, the backward method was used to select the variables.

The sensitivity, specificity, positive and negative predictive values, area under the curve and accuracy of all three models were computed in order to compare them. Two-thirds of the data were randomly chosen as the training set and the model sees and learns from them. The remaining one-third of data were considered as the test set. It is only used once a model is completely trained, and the test set is generally used to evaluate the competing model. All analyses were performed using software R 3.4.3 with the help of software packages including pROC, caret, e1071 and rminer. In this study, *p* < 0.05 was considered as the significance level.

## 3. Results

Of the 1141 participants, 590 (51.7%) were male and 551 (48.3%) were female. The result of angiography was that 750 (65.7%) were positive and 391 (34.3%) were negative. The frequency distribution of the demographic characteristics, other risk factors and the result of angiography are shown in Table 1. The results of fitting logistic regression model with backward method and also the results of the sensitivity analysis for naïve Bayes and SVM with radial kernel functions were used to determine the effective variables (Table 2). Seven, three and six variables were selected for logistic regression, naïve Bayes and SVM, respectively. For all three models, gender, age, and fast blood sugar (FBS) were common

In Table 3, the sensitivity, specificity, positive and negative predictive value, and area under the curve, are reported in order to compare the three models in their ability to predict the results of angiography.

**Figure 1:**
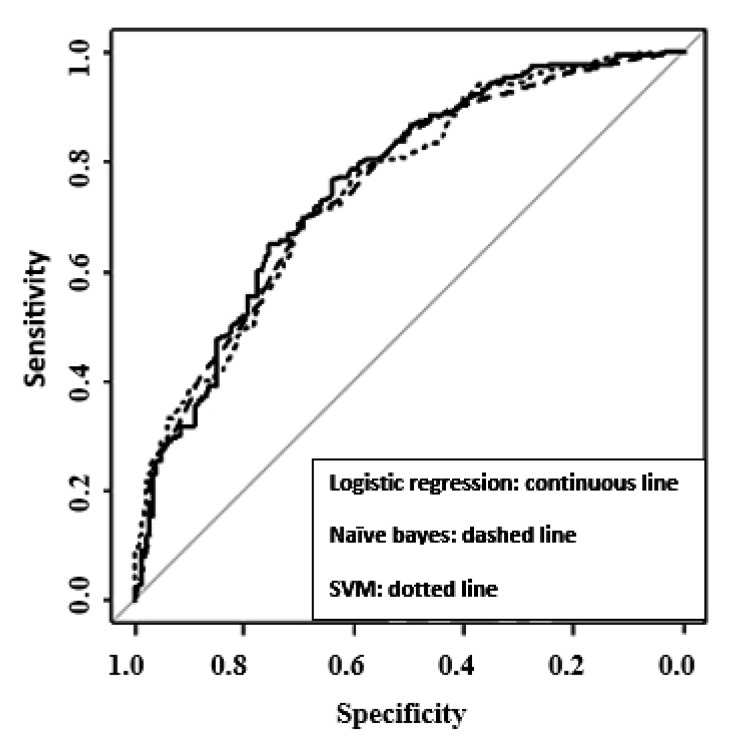
Receiver operating characteristic (Roc) curves for each of the models.

According to Table 3 and Figure 1, we compared the area under the curve of three models and found no significant difference for these values. Hence, a naïve Bayes model was selected as the best model because it can predict the result of the angiography correctly only with three variables. The SVM model with six predictor variables was second to the naïve Bayes model (Table 2).

## 4. Discussion

The objective of this study was firstly to use the naïve Bayes, SVM and regression models to predict the result of the angiography and then compare the performance of these models. Secondly, determining the factors that may predict the need for angiography. Determining these diagnostic factors may not only help in the management of cardiovascular disease, but may also reduce the need to perform an unnecessary coronary angiography by identifying people whose angiography result would be predicted to be negative, because coronary angiography is costly, invasive and accompanied by complications.

According to the results of the logistic regression, the age, gender, family history of kidney disease, history of myocardial infarction, history of heart disease, TG and FBG were statistically significant (*p* < 0.05) in predicting the results of an angiography. Nasab et al. have previously used a logistic regression model to investigate the important factors of cardiovascular disease for people over 35 years of age. Some variables that are the same as the present study in terms of affecting coronary artery disease are: sex, triglyceride, fasting blood glucose and age. While in both studies the family history of cardiovascular disease is not significant. Moreover, the serum HDL is not significant in the present study but it is significant in the report by Nasab et al., which may be due to the differences in age [11]. Furthermore, regarding naïve Bayes, gender, age and FBG, and for SVM gender, age, family history of kidney disease, history of myocardial disease, hypertension, and FBS were the most effective factors in determining the necessity of an angiography. In a study carried out by Heravi et al. to determine the risk factors of coronary arteries disease, some factors such as age, gender and diabetes presented a significant relationship with the number of involved vessels. These statistically significant factors being is consistent with the present study [12].

Miranda et al. used a naïve Bayes model to detect CVD risk in adults. The model exhibits a good performance for risk level detection of cardiovascular disease. Our results are compatible with this [13].

The SVM model using six predictor variables (gender, age, family history of kidney disease, history of myocardial infarction, high blood pressure, FBG) compared to the logistic regression model with seven predictor variables (gender–age–FBG–history of myocardial infarction–history of heart disease–TG–family history of kidney disease) predicts the result of angiography more accurately.

Mahmoodi used a combination of a fuzzy system and support vector machine classifiers to diagnose patients with heart disease. In this study, data of 270 people with 13 features (age, gender, chest pain, rest blood pressure, cholesterol, FBG, the result of angiography, old peak, slope, Thal, maximum heart rate, exercise-induced angina, number of major vessels colored by fluoroscopy) were used. In this study, the rates of categorization and sensitivity were considered as evaluation criteria with the percentage of 85% and 85.8%, respectively. According to the results, the combined system diagnosed patients with cardiovascular diseases with a high accuracy. This study shows that the use of SVM has as high as sensitivity compared to our research in which it is not combined with any other models [14].

Gonsalves et al. conducted research about the prediction of coronary heart disease (CHD). This research used the historical medical data to predict CHD by means of a machine learning (ML) algorithm: naïve Bayes, support vector machine and decision tree (DT), to find correlations in CHD data that might improve the prediction rate, and the data were extracted from the South African Heart Disease dataset of 462 cases. The empirical results report that the NB model is promising in terms of detecting CHD which is compatible with our results [15].

Ramalingam et al., used several machine learning algorithms such as support vector machine and naïve Bayes, to name but a few. It shows that the naïve Bayes model was computationally very fast and both the SVM and naïve Bayes performed extremely well [16].

Unnikrishnan et al., in order to predict the risk of CVD, used an SVM and showed that the SVM performed better than a logistic regression model [17]. In addition, Shafie et al. predicted coronary artery disease using bioinformatics algorithms. The results showed that SVM with 100% accuracy can distinguish between individuals with and without disease, which can be due to the ability of SVM to classify nonlinear classifications [18].

In terms of cardiovascular disease, several studies have been undertaken using SVM, naïve Bayes and logistic regression, but it should be taken into consideration that the performance of these models depends on numerous factors, including the size of the database, the number and type of predictor variables and the missing. Thus, selecting a model as the most efficient one is not reasonable.

## 5. Conclusions

As there was no significant difference among the models, gender, age, and FBG variables are the most important variables for predicting the result of angiography, because the naïve Bayes model was good at predicting it only with these three variables. In addition, these three variables were important in the other two models.

In addition, it may be necessary to give priority to patients with these characteristics that are more likely to have positive result for angiography so they can be treated more urgently.

## Figures and Tables

**Table 1:** Comparison of frequency of subjects’ demographics and risk factors based on the result of angiography.

**Variable**	**Angiography**		***p*-Value**
Age group	**Positive**	**Negative**	
18–39	21 (36.2)	37 (63.8)	(*p* < 0.001)
40–59	378 (61.0)	242 (39.0)	
60≤	351 (75.8)	112 (24.2)	
Levels of education			(*p* = 0.176)
Elementary	495 (64.3)	274 (35.7)	
Diploma	113 (71.5)	45 (28.5)	
Bachelor	111 (64.1)	62 (35.9)	
MA	31 (75.6)	10 (24.4)	
Gender			(*p* < 0.001)
Female	284 (51.5)	267 (48.5)	
Male	466 (78.9)	124 (21.1)	
Smoking Habit			(*p* < 0.001)
Smoker	184 (75.4)	60 (24.6)	
Used to smoke	119 (69.5)	52 (30.5)	
Non-smoker	447 (61.5)	279 (38.5)	
History of high blood pressure			(*p* = 0.303)
Yes	352 (67.3)	171 (32.7)	
No	398 (64.4)	220 (35.6)	
Family history of kidney disease			(*p* = 0.028)
Yes	208 (70.9)	85 (29.1)	
No	542 (63.9)	306 (36.1)	
History of cardiovascular disease			(*p* = 0.274)
Yes	337 (64.0)	189 (36.0)	
No	413 (67.1)	202 (32.9)	
History of myocardial infarction			(*p* < 0.001)
Yes	165 (81.6)	37 (18.4)	
No	585 (62.3)	354 (37.7)	
Family history of hypertension			(*p* = 0.001)
Yes	278 (60.0)	185 (40.0)	
No	472 (69.6)	206 (30.4)	
Fasting blood glucose			(*p* < 0.001)
Normal	253 (60.3)	166 (39.7)	
Prediabetes	164 (50.3)	162 (49.7)	
Diabetes	333 (84.0)	63 (16.0)	
Serum Triglycerides (mg/dL)			(*p* = 0.004)
Normal <150	487 (62.9)	287 (37.1)	
Borderline 150–199	148 (75.5)	48 (24.5)	
High >200	115 (67.2)	56 (32.8)	
Serum High density lipoprotein (mg/dL)			(*p* > 0.99)
Normal	282 (68.4)	130 (31.6)	
Risk range	468 (68.4)	216 (31.6)	

**Table 2:** The number of variables for each of the models.

Model	Number of Variables	Variables
Logistic regression	7	Gender–age–Fasting blood glucose–Triglyceride–Family history of kidney disease–History of cardiovascular disease–History of myocardial infarction
Naïve Bayes	3	Gender–age–FBG
Support Vector Machine	6	Gender–age–FBG–Family history of kidney disease–History of hypertension–History of myocardial infarction

Note. FBG, fasting blood glucose.

**Table 3:** Comparing the models.

Criteria	SVM	Naïve Bayes	Logistic Regression
Sensitivity	0.908	0.892	0.884
Specificity	0.401	0.428	0.442
Positive predictive values	0.706	0.712	0.715
Negative predictive values	0.737	0.715	0.707
Accuracy	0.71	0.713	0.713
AUC (Area Under Curve)	0.75	0.74	0.76
CI 95% (AUC)	0.70–0.80	0.70–0.80	0.71–0.80
